# Affective Temperaments, Panic Disorder and Their Bipolar Connections

**DOI:** 10.3390/medicina57030289

**Published:** 2021-03-19

**Authors:** Zsuzsanna Belteczki, Zoltan Rihmer, Sandor Rozsa, Julia Ujvari, Maurizio Pompili, Xenia Gonda, Péter Dome

**Affiliations:** 1 First Department of Psychiatry, University Hospital of Szabolcs-Szatmár-Bereg County, 4320 Nagykallo, Hungary; belteczkizsuzsa@gmail.com (Z.B.); julcsi0225@freemail.hu (J.U.); 2 Department of Psychiatry and Psychotherapy, Semmelweis University, 1085 Budapest, Hungary; rihmer.z@kronet.hu (Z.R.); gonda.xenia@med.semmelweis-univ.hu (X.G.); 3 Nyiro Gyula National Institute of Psychiatry and Addictions, 1135 Budapest, Hungary; 4 Department of Psychiatry, Washington University School of Medicine, St. Louis, MO 63110, USA; rozsaqqq@gmail.com; 5 Department of Neurosciences, Mental Health and Sensory Organs, Suicide Prevention Center, Sant’Andrea Hospital, Sapienza University of Rome, 00189 Rome, Italy; maurizio.pompili@uniroma1.it

**Keywords:** panic disorder, bipolar disorder, agoraphobia, affective temperaments, comorbidity, TEMPS

## Abstract

*Background and Objectives:* The role of affective temperament in the genesis and outcome of major mood disorders is well studied, but there are only a few reports on the relationship between panic disorder (PD) and affective temperaments. Accordingly, we aimed to study the distribution of affective temperaments (depressive (DE); cyclothymic (CT); irritable (IRR); hyperthymic (HT) and anxious (ANX)) among outpatients with PD. *Materials and Methods:* Affective temperaments of 118 PD outpatients (80 females and 38 males) with or without agoraphobia but without any other psychiatric disorder at the time of inclusion were evaluated using the Temperament Evaluation of the Memphis, Pisa, Paris, and San Diego Autoquestionnaire (TEMPS-A) and compared with the affective temperament scores of control subjects. All patients were followed up for at least 1.5 years in order to detect the onset of any major affective disorders, substance use disorders and suicide attempts. *Results:* Among females, the dominant ANX and DE temperaments were four and three times as common as in a large normative Hungarian sample (for both cases *p* < 0.01). Among male PD patients, only the dominant DE temperament was slightly overrepresented in a non-significant manner. Females with PD obtained significantly higher scores on ANX, DE and CT subscales of the TEMPS-A, whereas males with PD showed significantly higher scores on ANX, DE and HT temperament subscales compared with the members of a large normative Hungarian sample and also with a gender- and age-matched control group. During the follow-up, newly developed unipolar major depression and bipolar spectrum (bipolar I or II and cyclothymic) disorders appeared in 64% and 22% of subjects, respectively. *Conclusions:* Our preliminary findings suggest that a specific, ANX-DE-CT affective temperament profile is characteristic primarily for female patients, and an ANX-DE-HT affective temperament profile is characteristic for male patients with PD, respectively. These findings are in line with expectations because PD is an anxiety disorder par excellence on the one hand, whereas, on the other hand, it is quite frequently comorbid with mood (including bipolar) disorders.

## 1. Introduction

Panic disorder (PD) is an anxiety disorder characterized by repeated panic attacks, frequently accompanied by anticipatory anxiety and agoraphobia. Epidemiological studies from different parts of the world show that the lifetime prevalence of PD is between 1% and 4.8%, and its lifetime, one-year and past-month prevalences in Hungary are 4.4%, 3.1% and 2.0%, respectively [1,2,3]. In the majority of cases, PD is comorbid with another psychiatric diagnosis. During the long-term course of PD, major depressive episodes or bipolar disorder are the most common comorbid conditions, ranging from 30% to 90% [4,5,6,7,8,9,10]. Mood disorders can develop intra-episodically (simultaneously) and/or longitudinally. These comorbid conditions can result in a decreased treatment response and make the long-term prognosis worse [1,2,6,11,12,13].

PD is commonly associated with bipolar (particularly bipolar II) disorder and, on the other hand, in patients with bipolar disorder the most frequent comorbid anxiety disorder is PD, with a 15–20% lifetime prevalence, whereas the same rate in unipolar depression is around 10% [4,5,14,15,16]. The strong relationship between panic and bipolar disorder can also be demonstrated in family studies. PD is much more common among the first-degree relatives of bipolar patients than in the relatives of unipolar depressive ones [5,14,17].

Affective temperament is considered to carry the temporally steady “core” of personality and contributes to setting an individual’s moods, activity level and rhythms, as well as their variability, whereas personality, a broader phenotype, also refers to acquired characterological determinants and interpersonal operations. It is already manifested in childhood and is partially genetically determined and remains relatively stable over time [18,19,20,21,22,23,24].

Affective temperaments also carry the advantages and disadvantages of emotional reactivity; although they do not pose a problem in mild forms, extreme variants predispose people to some forms of psychiatric disorders and—with the exception of those with a hyperthymic temperament—to suicidal behavior [23,25,26,27,28]. Based on this concept, an instrument called the Temperament Evaluation of the Memphis, Pisa, Paris, and San Diego Autoquestionnaire (TEMPS-A) has been developed for assessing depressive (DE), hyperthymic (HT), cyclothymic (CT), anxious (ANX) and irritable (IRR) temperaments. TEMPS-A contains 110 (109 for males) simple yes-or-no items [19,20,23,29]. Affective temperaments are the subsyndromal forms and frequently the harbingers of mood disorders and they also have an important role in the development of minor and major mood disorders, including the direction of the polarity of the episodes and the symptom-formation of the acute clinical picture. Furthermore, they can considerably influence the long-term course and outcome, including treatment responsiveness, compliance and suicidality [18,19,20,23,27,28,30].

Although the relationship between affective temperament and major (unipolar and bipolar) mood disorder is well studied [18,23,26,27,30,31,32,33,34], less is known about the relationship between affective temperament and anxiety, particularly PD [5,15]. The aim of this study was to investigate the specific affective temperaments in outpatients with PD. As unipolar depressives typically have a DE temperament and bipolar patients exhibit mostly HT or CT features [23], we hypothesized that patients with panic (anxiety) disorder would show a predominantly ANX affective temperament but because of the frequent depressive and bipolar comorbidities, the presence of DE, HT and CT characteristics may also be expected to some degree.

## 2. Materials and Methods

One hundred and eighteen (80 female and 38 male) DSM-5-diagnosed PD patients with or without agoraphobia but without any other major psychiatric disorders (see exclusion criteria below) who visited our adult outpatient department between 30 September 2016 and 30 September 2017 were included. All patients were followed up until 31 March 2019 in order to detect the onset of any major affective disorders, substance use disorders and suicide attempts. To determine the affective temperaments we used the Hungarian version of TEMPS-A [19,20,29]. The Chronbach alpha coefficients for the temperament scales were quite high: depressive 0.76, cyclothymic 0.84, hyperthymic 0.84, irritable 0.78 and anxious 0.88.

The distribution of affective temperaments (i.e., the rate of “dominant” affective temperaments; defined below) and the data on cross-sectional and lifetime comorbidity (agoraphobia, major depression, bipolar I bipolar II and cyclothymic disorder, addictive disorder, suicide attempts) and family history data for first- and second-degree relatives in regard to affective, anxiety and substance-use disorders and completed suicide were gathered and analyzed. Affective temperaments were determined according to their z-scores in each subscale. An affective temperament was considered to be manifested as “dominant” if its z-score was at least 2 standard deviations (SD) above the z-score of the normative sample [29].

The exclusion criteria at intake were current or previous psychotic disorders, unipolar depression, bipolar and bipolar spectrum disorders, OCD, GAD, other anxiety disorders (except for PD and/or agoraphobia), dementia and concomitant severe somatic diseases. Altogether, fifty-seven patients (40 females and 17 males; mean age: 48.5 years) were excluded at study entry because they had lifetime comorbid psychiatric disorder(s) other than agoraphobia. In detail, 29 and 28 subjects were excluded due to bipolar disorders and unipolar major depression, respectively (12% of them also had a comorbid substance-use disorder). Thus, the total number of contacted patients was 175 (118 included and 57 excluded subjects) in our study.

The presence of current psychiatric disorders was considered based on the clinical interviews with patients. The presence of previous psychiatric disorders was established based on the medical history (i.e., on patient documentation and patients’ own reports). Participants were not under guardianship and, since we excluded subjects with dementia, the participants’ ability to consent was retained.

The data from the study group were statistically compared with normative data for the Hungarian population obtained from the study by Rózsa et al. (2008), which validated the TEMPS-A in Hungary [29]. Furthermore, the 118 cases were also frequency-matched by age and sex to 118 controls from the sample of the abovementioned validation study by Rózsa et al. (2008) [29]. A similar comparison was conducted between those 23 PD patients who had remained free from any comorbid major affective disorders during the follow-up and an age- and gender-matched control group, of which the 23 members were chosen from the sample of the validation study by Rózsa et al. (2008) [29].

The statistical comparison was performed using a two-sample *t*-test for continuous variables, and Chi-squared statistics for categorial variables. The alpha level was set at 5%. Statistical analysis was done with SPSS20. Participation was voluntary and anonymous and patients gave their written informed consent to participate in the study. The study was approved by the local ethical board of the University Hospital of Szabolcs-Szatmár-Bereg County, Nyíregyháza, Hungary.

## 3. Results

Out of the 118 PD patients, 80 (68%) were females and 38 (32%) males and the average age at inclusion was 44.4 years (range: 19–73 years) (Table 1). The average duration of PD was 10.1 years (ranging from 0.2 to 26 years). Seventy-seven patients (60 females and 17 males) had experienced agoraphobia at baseline and 10 patients (6 females and 4 males) experienced newly developed agoraphobia during the 1.5–2.5-year follow-up period (Table 1). Six patients (5%) had made a prior suicide attempt. During the follow-up, newly developed unipolar major depression, bipolar spectrum disorder (bipolar I or II and cyclothymic disorders) and substance-use disorders were observed in 64%, 22% and 9.5% of patients, respectively. The majority of patients with newly developed bipolar spectrum disorders had bipolar II disorder (19/26 = 73%). None of the patients made a suicide attempt during the follow-up. The family history of any anxiety and major affective disorders among the first- and second-degree relatives was 71% and the same rate for substance-use disorders was 22%. Thirteen percent of patients reported completed suicide among first- and second-degree relatives (Table 1).

Among females the “dominant” ANX and DE temperaments were four and three times as common as in the large normative Hungarian sample, whereas differences for the remaining three temperament types were not significant (Table 2). Corresponding comparisons in males did not yield significant differences.

Females with PD obtained significantly higher scores on ANX, DE and CT subscales of the TEMPS-A, whereas males with PD showed significantly higher scores on ANX, DE and HT subscales compared to the gender- and age-matched control group and the large normative Hungarian sample as well (Table 3).

In comparison with age-matched control subjects, females with “pure” PD (i.e., females with PD but without the onset of a comorbid major affective disorder during the follow-up) scored significantly higher on the ANX and CT subscales (*p* < 0.001 and *p* < 0.05, respectively), whereas males with “pure” PD scored significantly higher on the HT subscale (*p* < 0.05). These results are similar to those from the total patient sample, but for both genders some of the findings from the total sample could not be detected here (these differences may be explained by the low number of subjects with “pure” PD (*n* = 23)).

## 4. Discussion

We sought to assess temperaments in patients suffering from PD. In our sample of PD outpatients we found that the female predominance; the clinical and family history characteristics; the high rate of major affective disorders and substance-use disorder comorbidities; as well as the high family loading for mood, anxiety and substance-use disorders and suicidal behavior; were in agreement with previous studies [4,5,6,7,11,12,14,15,17,35]. Our findings on the high rates of newly diagnosed unipolar major depression (64%) and bipolar spectrum disorders (22%) during the follow-up period corroborated the well-established high comorbidity of panic and mood disorders [5,15,35]. If we consider all contacted patients (i.e., the 118 included and 57 excluded subjects), the rate of lifetime panic-bipolar disorder comorbidity is about 31.5% (55/175). This rate is about ten-times higher than the lifetime prevalence of bipolar I and II disorders in the general population [36], which further supports the strong relationship between panic and bipolar disorders.

A meta-analysis of anxiety disorders and temperament investigated only temperament dimensions (novelty seeking, harm avoidance, reward dependence and persistence) determined by the psychobiological model of Cloninger and found that, like patients with other anxiety disorders, subjects with PD show a marked and state-dependent harm-avoidant behavioral pattern [37,38]. However, the literature on affective temperament according to the model of Akiskal et al. [19,20] in PD is scarce, and we were able to find only a few prior studies on this subject. Comparing affective temperament profile, as measured by the TEMPS-A, of 42 PD and 44 OCD patients in Turkey, Fistikci et al. found that the dominant DE temperament was more common in the OCD group, whereas HT temperament scores were higher in the PD group [13]. However, no comparison with normal controls was reported in that study. Investigating the relationship between PD, affective temperaments and impulsivity, another study from Turkey found that the 70 enrolled PD patients had significantly higher scores on the DE, CT, IRR and ANX subscales of TEMPS-A and had also higher scores on attentional impulsivity than that the 58 healthy controls [39]. A third study from Turkey corroborated that DE, CT, IRR and ANX scores were higher for PD patients (*n* = 60) than those for age-, gender- and education level-matched control subjects (*n* = 37), whereas HT scores did not differ significantly between patients and controls [40]. An Italian study found the same pattern—i.e., except for HT, scores for the remaining four temperaments were significantly higher for PD patients (*n* = 64) than for healthy controls (*n* = 44) [41,42]. Similarly a very recent study from Germany reported that among patients with PD (*n* = 109; 34 out of them had comorbid major depression), DE, CT, IRR and ANX scores were elevated and HT scores were lower when compared to healthy controls (*n* = 536) [43].

The number of PD patients and control subjects included in our study exceeds the number of PD patients and controls in any previous studies on this topic. Considering our main findings, we were able to conclude that a specific ANX-DE-CT affective temperament profile is characteristic for female and an ANX-DE-HT pattern is characteristic for male patients with PD. This is an expected finding because PD belongs to anxiety disorders and it is quite frequently comorbid with unipolar depression and bipolar disorder [7,8,15,35]. These results support and extend the horizon of the specific pathoplastic role of affective temperaments in different psychiatric disorders. We know that HT and CT temperaments are characteristic for bipolar I patients, CT for bipolar II patients and DE for unipolar depressive subjects [23]. The findings presented here indicate that the affective temperament profile of PD patients is somewhat different from those with unipolar or bipolar major mood disorders because the prevailing temperament types in PD are the ANX and DE ones, whereas the elevated CT scores for females and HT scores for males might be related to the frequent bipolar comorbidity in PD [15]. The finding that a specific ANX-DE-CT affective temperament profile is characteristic primarily for female PD patients, along with ANX-DE-HT for male PD patients, is in accordance with the finding that in the general population CT is more common in females and HT is characteristic primarily for males [23]. Finally, our main finding that, in contrast to unipolar major depressive and bipolar disorders, ANX is the chief affective component of PD supports the view of Akiskal, who has added the fifth—namely, the anxious temperament—to the four Kraepelinian affective temperaments [18,19,20].

This preliminary study has several limitations. First and foremost, the relatively small sample size (especially for males) impairs the opportunity to generalize our results. Another major limitation is that current (i.e., at the time of the inclusion) and lifetime diagnoses were not based on structured interviews (such as SCID) and that in the majority of patients a comorbid mood disorder began during the follow-up period. Although determining the proportion of PD patients with the later development of one (or more) psychiatric comorbidities was not a primary objective of this study, the relative shortness of the follow-up period is another limitation of the study. Despite these shortcomings, our results are in good agreement with the results of relevant previous studies on this topic [39,40,41,43,44]. Accordingly, both previous investigations and our current study have found that ANX and DE temperament types are associated with PD. At the same time, unlike in the majority of previous studies, in our sample, the IRR temperament was not associated with PD but higher HT temperament scores (in males) were associated with PD. In addition, in the majority of prior studies higher CT temperament scores were associated with PD but in our sample this association was found only among females.

## 5. Conclusions

Our preliminary findings suggest that a specific ANX-DE-CT affective temperament profile is characteristic primarily for females with PD and an ANX-DE-HT affective temperament profile is characteristic primarily for male patients with PD. These findings are in line with expectations, because PD is an anxiety disorder *par excellence* on the one hand, whereas, on the other hand, it is quite frequently comorbid with mood (including bipolar) disorders.

## Figures and Tables

**Table 1:** Demographic, clinical and family history data of our 118 panic disorder outpatients.

	Females	Males	Total
Number of patients (%)	80 (68%)	38 (32%)	118 (100%)
Average age in years (range)	44.82 (22–60)	44.07 (19–73)	44.44 (19–73)
Duration of panic disorder (PD) in years (range)	10.8 (0.2–26)	9.5 (0.3–25)	10.1 (0.2–26)
Comorbidity at baseline			
Agoraphobia, *n* (%)	60 (75%)	17 (45%)	77 (65%)
Comorbidity at follow-up			
Agoraphobia, *n* (%)	6 (7.5%)	4 (10%)	10 (8.5%)
Unipolar major depression, *n* (%)	55 (69%)	20 (53%)	75 (64%)
Substance use disorder, *n* (%)	3 (37.5%)	8 (21%)	11 (9.5%)
Bipolar disorder and cyclothymia, *n* (%)	17 (21%)	9 (24%)	26 (22%)
Previous suicide attempts, *n* (%)	4 (5%)	2 (5%)	6 (5%)
Family history (1- and 2-degree relatives) for anxiety and/or affective disorder *n* (%)	54 (67.5%)	30 (79%)	84 (71%)
Family history (1- and 2-degree relatives) for substance-use disorder, *n* (%)	18 (23%)	9 (24%)	26 (22%)
Family history (1- and 2-degree relatives) for completed suicide, *n* (%)	8 (10%)	8 (21%)	16 (13.5%)
Mean length of follow-up in month (SD)	26.3 (3.7)	26.7 (3.7)	26.4 (3.7)

**Table 2:** Distribution of dominant affective temperaments (average score + 2 standard deviations (SD)) in our PD sample and in a large normative Hungarian population—Females.

Temperaments	Female Patients (*n* = 80)		Normative Hungarian Sample, Females (*n* = 797 [29])		Difference (*p*, χ^2^ Test)
	N	%	N	%	
Depressive	8	10	27	3.4	2.9× (*p* < 0.01)
Cyclothymic	5	6.3	28	3.5	1.8× (not significant)
Hyperthymic	1	1.3	16	2.0	0.65× (not significant)
Irritable	0	0	15	1.9	-
Anxious	16	20	38	4.8	4.2× (*p* < 0.0001)

**Table 3:** Temperament Evaluation of the Memphis, Pisa, Paris, and San Diego Autoquestionnaire (TEMPS-A) subscale scores (means (M); standard deviations (SD)) and their comparisons (differences; *t*-test) compared between panic disorder patients (PD), age- and gender-matched controls (MC) and members of a large normative Hungarian sample (NHS).

Temperaments	Panic Disorder (PD) Patients (*n* = 118)		Age- and Gender-Matched Controls (MC) (*n* = 118)		Normative Hungarian Sample (NHS) (*n* = 1132) [29]		Difference PD vs. MC (*p*, *t*-Test)		Difference PD vs. NHS (*p*, *t*-Test)	
	Females M (SD)	Males M (SD)	Females M (SD)	Males M (SD)	Females M (SD)	Males M (SD)	Females	Males	Females	Males
Depressive	0.49 (0.17)	0.39 (0.17)	0.39 (0.17)	0.31 (0.14)	0.33 (0.15)	0.31 (0.15)	*p* < 0.001	*p* < 0.05	*p* < 0.001	*p* < 0.01
Cyclothymic	0.39 (0.25)	0.35 (0.22)	0.28 (0.20)	0.28 (0.24)	0.35 (0.20)	0.33 (0.22)	*p* < 0.01	n.s.	*p* < 0.05	n.s.
Hyperthymic	0.46 (0.20)	0.60 (0.24)	0.42 (0.20)	0.48 (0.20)	0.47 (0.20)	0.53 (0.22)	n.s.	*p* < 0.05	n.s.	*p* < 0.05
Irritable	0.28 (0.17)	0.30 (0.19)	0.23 (0.17)	0.30 (0.22)	0.29 (0.18)	0.31 (0.20)	n.s.	n.s.	n.s.	n.s.
Anxious	0.50 (0.22)	0.32 (0.19)	0.32 (0.19)	0.18 (0.16)	0.30 (0.18)	0.20 (0.16)	*p* < 0.001	*p* < 0.001	*p* < 0.001	*p* < 0.001

## Data Availability

The raw dataset of the current study is available from the corresponding author on reasonable request.
